# Physical Dormancy Release in *Medicago truncatula* Seeds Is Related to Environmental Variations

**DOI:** 10.3390/plants9040503

**Published:** 2020-04-14

**Authors:** Juan Pablo Renzi, Martin Duchoslav, Jan Brus, Iveta Hradilová, Vilém Pechanec, Tadeáš Václavek, Jitka Machalová, Karel Hron, Jerome Verdier, Petr Smýkal

**Affiliations:** 1 Instituto Nacional de Tecnología Agropecuaria, Hilario Ascasubi 8142, Argentina; renzipugni.juan@inta.gob.ar; 2 Department of Botany, Palacký University, Šlechtitelů 27, 783 71 Olomouc, Czech Republic; martin.duchoslav@upol.cz (M.D.); HradilovaI@seznam.cz (I.H.); 3 Department of Geoinformatics, Palacký University, 17. listopadu 50, 771 46 Olomouc, Czech Republic; jan.brus@upol.cz (J.B.); vilem.pechanec@upol.cz (V.P.); 4 Department of Mathematical Analysis and Applications of Mathematics, Palacký University, 17. listopadu 12, 771 46 Olomouc, Czech Republic; TadeasVaclavek@seznam.cz (T.V.); jitka.machalova@upol.cz (J.M.); karel.hron@upol.cz (K.H.); 5 UMR 1345 Institut de Recherche en Horticulture et Semences, Agrocampus Ouest, INRA, Université d’Angers, SFR 4207 QUASAV, 49070 Beaucouzé, France; jerome.verdier@inrae.fr

**Keywords:** association mapping, climate adaptation, germination, genomics, legumes, *Medicago*, plasticity, physical dormancy, seed dormancy

## Abstract

Seed dormancy and timing of its release is an important developmental transition determining the survival of individuals, populations, and species in variable environments. *Medicago truncatula* was used as a model to study physical seed dormancy at the ecological and genetics level. The effect of alternating temperatures, as one of the causes releasing physical seed dormancy, was tested in 178 *M. truncatula* accessions over three years. Several coefficients of dormancy release were related to environmental variables. Dormancy varied greatly (4–100%) across accessions as well as year of experiment. We observed overall higher physical dormancy release under more alternating temperatures (35/15 °C) in comparison with less alternating ones (25/15 °C). Accessions from more arid climates released dormancy under higher experimental temperature alternations more than accessions originating from less arid environments. The plasticity of physical dormancy can probably distribute the germination through the year and act as a bet-hedging strategy in arid environments. On the other hand, a slight increase in physical dormancy was observed in accessions from environments with higher among-season temperature variation. Genome-wide association analysis identified 136 candidate genes related to secondary metabolite synthesis, hormone regulation, and modification of the cell wall. The activity of these genes might mediate seed coat permeability and, ultimately, imbibition and germination.

## 1. Introduction

Plant species exhibit a high ability for local adaptation and phenotypic plasticity that may contribute to their distribution range. While local adaptation is the genetically fixed advantage of a population under certain environmental conditions [1], phenotypic plasticity is the ability of a genotype to generate different phenotypes in response to variation in the environment [2,3]. This variation is created by mutation, recombination, and introgression, and by population genetics processes, such as genetic drift and natural selection, that determine its evolutionary fate. Understanding of the genetic basis of local adaptation and phenotype plasticity is relevant to climate change, crop production, conservation, and understanding of speciation. The combination of genomics and ecology enables genome-wide analysis to reveal the interaction between organisms and environment [4] and to identify genomic regions involved in adaptation [5]. On the other hand, phenotypic plasticity may allow species to grow and survive in different environments despite a restricted genetic base. Thus, phenotypic plasticity could be advantageous under variable environments, including climatic change [6], and may also increase species invasion success [7].

Timing of seed germination is one of the key steps in plant life, influencing the subsequent destiny of individuals as well as whole populations at determined area. Plants have evolved various mechanisms to control seed germination within- and among-seasons and in relation to the diversity of climates, habitats, and biotic pressures [8,9]. Three different kinds of dormancy have been described to allow optimal germination timing under specific environmental conditions [8,9]: (1) morphological dormancy (MD) refers to seeds that have an underdeveloped embryo and require time to grow; (2) physiological dormancy (PD) prevents embryo growth and seed germination until chemical changes occur, involving abscisic acid and gibberellins metabolism, among other factors; and (3) physical dormancy (PY) is caused by water-impermeable palisade cells in the seed coat. PY occurs in at least 18 Angiosperm families and is frequent in legumes [10,11,12].

Adaptation to the local environment operates through selection for successful germination and early plant establishment [13]. The prevention of germination of a certain proportion of seeds even under optimal conditions for germination reduces the risk of mortality in less predictable environmental conditions. It has been suggested theoretically [14] and shown empirically [15] that adaptation for dormancy is a bet-hedging strategy to magnify the evolutionary effect of “good” years and to dampen the effect of “bad” years, i.e., to buffer environmental variability [16]. In addition, species that frequently and reliably produce seed can afford riskier germination under unfavorable conditions (e.g., small rainfall events) because the consequences of failure to establish are less dire than for species that do not reliably produce seed [17]. Desert annuals that do not frequently and reliably reproduce are model organisms for the study of the bet-hedging strategy [18].

In order to germinate, specific environmental conditions need to be met to break the seed dormancy [8]. However, less is known about the factors which release the PY dormancy. Through experimental studies, it was shown that, in addition to scarification, wet or dry heat were found to be effective [8,10,19]. In addition, natural conditions, such as temperature and soil moisture oscillations, are the major players [20,21]. Laboratory studies have demonstrated an association between seed responsiveness to temperature and environmental thermic characteristics [10,22,23]. However, only limited data are available on how and why PY varies inter- and intra-specifically in natural ecosystems [24]. Legumes are thus a model example for studies of PY dormancy patterns in relation to environmental variations. The study of Rubio de Casas et al. [25] showed a latitudinal gradient in PY dormancy in legumes. Thus, PY dormancy increases from regions with long growing seasons (e.g., tropical climate) in lower latitudes to regions with a seasonal climate in higher latitudes. However, there are some studies of intraspecific PY dormancy variation along environmental gradients in several legume species [10,26,27,28,29] that are in disagreement with the results of Rubio de Casas et al. [25]. *Medicago truncatula* (barrel medic) is an annual, diploid, self-fertile species with a natural geographic distribution across the Mediterranean Basin. Phenotypic variation among populations has been explained by the adaptation to local environmental conditions [30]. *M. truncatula* offers an excellent model to study seed dormancy in relation to genetic and environmental factors because within its range it inhabits environments with rather contrasting climatic conditions, differing not only in mean annual temperature and precipitation, but also in within- and across-season variability (unpredictability). Its seeds exhibit both physical and physiological dormancy. Physiological dormancy in *M. truncatula* seeds is non-deep, and is removed during the seed ripening period [31,32]. The short after-ripening period to overcome PD (<3 months) determines that PY release is the most important trait to regulate the timing of seedling emergence. Despite this, most germination studies in *M. truncatula* eliminate the influence of PY dormancy through prolonged periods of storage (>9 months) and/or by seed scarification [33,34]. Large georeferenced collections, a reference genome, and a high-density single nucleotide polymorphism (SNP) map of more than 260 genotypes of *M. truncatula* are available [30,35] and were used for study of the association between the genome and the environment in relation to flowering [35,36,37]. We have taken advantage of this georeferenced collection to analyze the patterns of dormancy release in 178 accessions of *M. truncatula* originating from various environments in the Mediterranean basis and tested seeds under alternating temperatures. The following questions were addressed: (i) Is there variation in physical seed dormancy among accessions to temperature treatments? (ii) Which of the ecological factors acting as potential adaptation drivers are correlated with dormancy? (iii) Are there any candidate genes that might be related to seed dormancy release in *Medicago*, using genome-wide association (GWAS) analysis?

Our study showed that phenotypic plasticity of final dormancy was significantly correlated with increased aridity, suggesting that plastic responses to external stimuli provide seeds with strong bet-hedging capacity and the potential to cope with high levels of environmental heterogeneity. Genome-wide association analysis performed on seven seed dormancy traits and three bioclimatic variables identified 136 candidate genes as potential regulators of physical dormancy. A large proportion of candidate genes were annotated as involved in synthesis of secondary metabolites, in cell wall modification, and hormone regulation. The knowledge about the regulation of seed dormancy by environmental factors could be extended to other legume species, particularly to crop wild relatives of economically important crops, such as chickpea, lentil, and faba bean. In addition, it can be used in a conservation biology context for the management of endangered plant species in relation to climate change.

## 2. Results

### 2.1. Responses of Dormancy Traits of Medicago Accessions to Experimental Temperature Treatments

**Figure 1:**
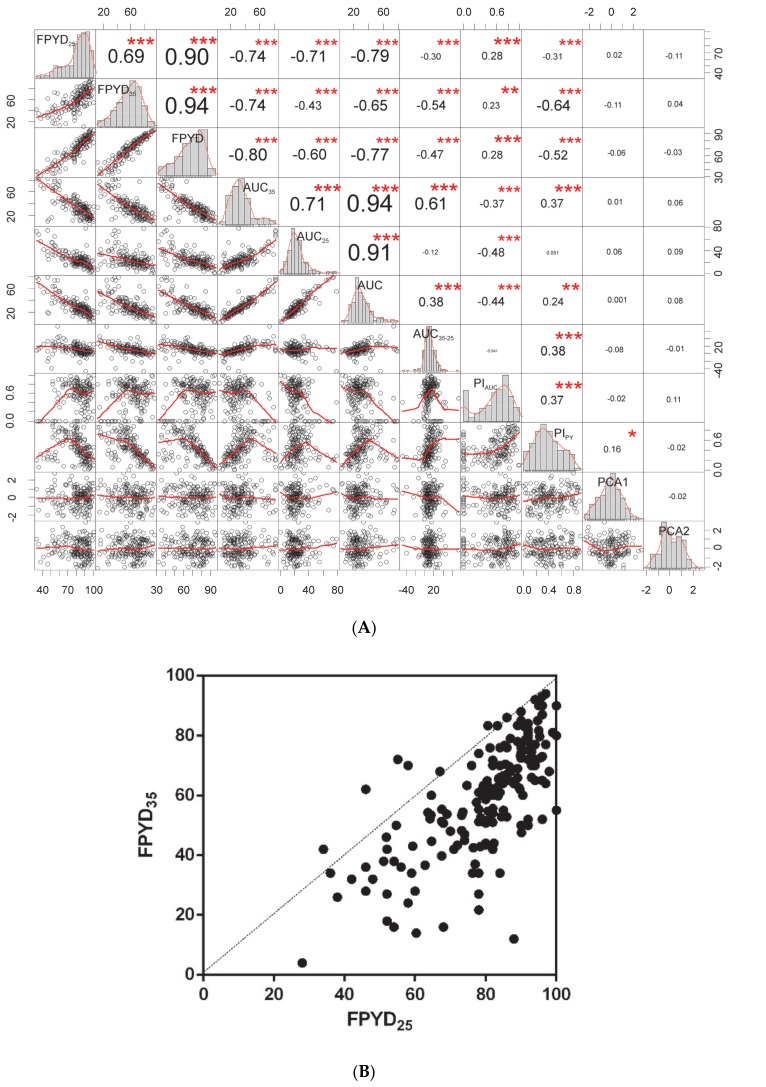
Correlations among dormancy release traits. (**A**) Correlation chart of dormancy release traits and ordination scores of environmental principal component analysis, PCA (first two ordination axes; PCA1, PCA2; see Figure 2A,B). The distribution of each variable is shown on the diagonal. Bellow the diagonal the bivariate scatter plots with a fitted smooth line (loess) are displayed. Above the diagonal the value of the Pearson correlation coefficient plus the significance level as stars are displayed (* *p* ≤ 0.05, ** *p* ≤ 0.01, *** *p* ≤ 0.001). (**B**) Relationship between final physical dormancy (PY) dormancy of each accession under two temperature treatments (FPYD_35_ and FPYD_25_).

Most dormancy traits exhibited a near normal distribution, and a wide range of variability (Figure 1A; Supplementary data Figure S1). Final PY dormancy (FPYD), a proportion of dormant seeds after 88 days of incubation onto water-saturates, ranged from 34% to 100% with mean 80% (SD = 15) at 25/15 °C treatment, and from 4% to 94% with mean 60% (SD = 19) at 35/15 °C treatment. Comparison of responses of each accession to two temperature treatments showed a remarkable effect of larger temperature alternation on dormancy release in a majority of accessions (Figure 1B). The germination pattern (area under curve, AUC_M_) ranged from 3 to 79, and, similarly to FPYD, larger temperature alternation increased the dormancy release (AUC_25_: mean ± SD 24 ± 13, range 0–79; AUC_35_: 34 ± 17, range 5–82), except for some accessions (16%) where the differential (AUC_35-25_) was negative (Figure 1A and Figure S1). Both phenotypic plasticity indexes, based on the minimum and the maximum value among the two temperature treatments divided by the maximum value (PI), showed large ranges with mean PI_AUC_ being slightly higher (0.56 ± 0.29, range 0.00–1.00) than mean PI_PY_ (0.43 ± 0.23, range 0.00–0.91) (Figure 1A and Figure S1). All dormancy traits were moderately to strongly correlated (up to |0.75|, excluding FPYD_M_ and AUC_M_ with some correlations up to |0.94|), except for PI_AUC_, which was significantly correlated only with AUC_35_ and AUC_25_ (Figure 1A; Supplementary data Figure S2, S3).

### 2.2. Associations of Environmental Gradients with Dormancy Traits of Medicago Accessions

**Figure 2:**
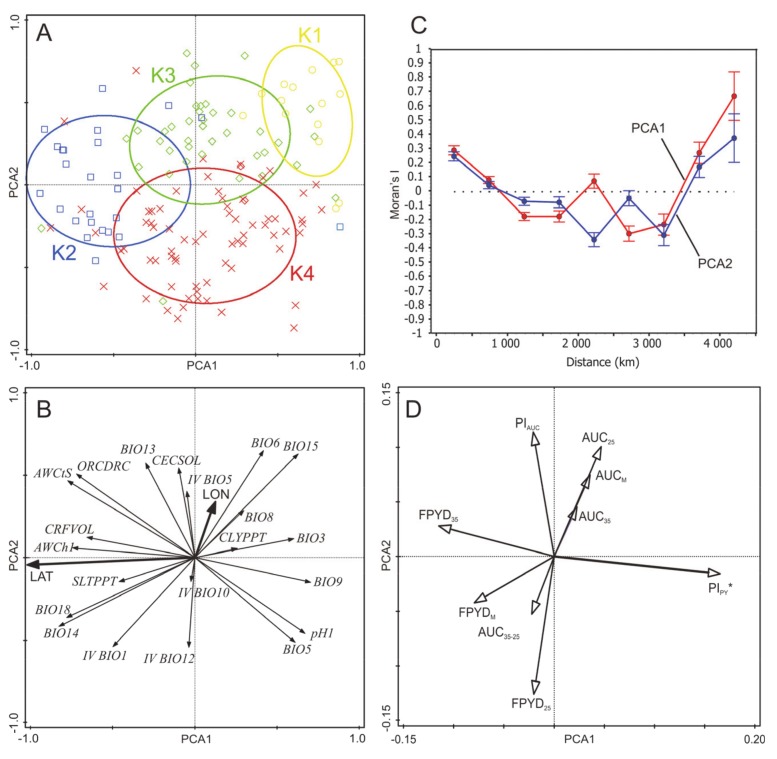
Principal component analysis (PCA) of selected bioclimatic and soil variables of *Medicago* accessions and multiple correlations of dormancy traits with ordination axes. (**A**,**B**) Principal component analysis (PCA) of selected bioclimatic and soil variables of Medicago accessions. Each accession is classified according to cluster analysis of environmental variables into one of four clusters (see Methods). The ellipses were created based on a model of bivariate normal distribution of the cluster class symbols (estimated from a variance–covariance matrix of their X and Y coordinates) to cover 95% of that distribution’ cases. A comparison of selected environmental variables among clusters is shown in Supplementary fileSupplementary file Tables S1 and S2. Vectors of geographic variables (latitude, longitude) were added into the diagram after PCA to visualize spatial gradients of environment. Variables BIO14 and 18 were log(x+1) transformed before analyses. (**C**) Spatial autocorrelation diagram of Moran’s I for the first two ordination axes of PCA (PCA1, PCA2). Mean ± 95% CI of I for respective distance class is calculated. (**D**) Multiple correlations of dormancy traits with the first and the second ordination axes of the environmental PCA. Each arrow points in the direction of the steepest increase of the values for corresponding dormancy trait. The angle between arrows indicates the sign of the correlation between the variables. The length of the variable arrows is the multiple correlation of that variable with the ordination axes. Dormancy trait significantly correlated (*p* ≤ 0.05, spatial correlation) with any ordination axis has an asterisk.

Principal component analysis (PCA) of a reduced data set containing 14 climatic and eight soil variables revealed two clear environmental gradients (Figure 2A,B). The first ordination axis explained 30.4% of the total variation and can be interpreted as the gradient of aridity that is tightly correlated with latitude (i.e., the north–south gradient). Climatic variables with the highest positive/negative correlation with the fist ordination axis represent temperatures of the warmest month (BIO5; Pearson’s r = 0.61 ***) and the driest quarter (BIO9, r = 0.71 ***), isothermality (BIO3, r = 0.59 ***), precipitation of the driest month (BIO14; r = −0.83 ***) and precipitation of the warmest quarter (BIO18, r = −0.78 ***). Concerning soil variables, pH index is positively correlated (r = −0.69 ***) while soil organic carbon content (ORCDRC, r = −0.75 ***), available soil water capacity (AWCh1, r = −0.75 ***), and saturated water content (AWCtS, r = −0.79 ***) are negatively correlated with the first axis. Latitude (r = −0.77 ***) but not longitude (r = 0.07) is strongly negatively correlated with the first axis. The second ordination axis explained 17.3% of the total variation and can be interpreted as combined gradient of seasonality and inter-annual variability, with weak geographic (i.e., west–east) trend (latitude: r = −0.01, longitude: r = 0.24 ***). The most correlated variables with the second axis were precipitation seasonality (BIO15, r = 0.61 ***) and minimal temperature of the coldest month (BIO6, r = 0.63 ***), but inter-annual variability of temperature (IV BIO1, r = −0.54 ***) and precipitation (IV BIO12, r = −0.54 ***) also had high correlation coefficients. Both synthetic environmental variables (PC1, PC2) were spatially structured as revealed by Moran’s I correlogram (both *p* < 0.001), showing positive autocorrelation at short and large distance classes and negative autocorrelation at intermediate distance classes (Figure 2C). Inspection of dormancy trait correlations with ordination axes representing synthetic environmental variables showed that only PI_PY_ was significantly correlated with the first ordination axis (r = 0.16 *), even after correction for spatial autocorrelation (*p* = 0.032). Other dormancy traits did not show any significant correlation with the first two ordination axes of PCA (Figure 2D; Supplementary data Figure S2). Neither dormancy trait showed any spatial autocorrelation (all Moran’s I correlograms had *p* > 0.40, not shown).

Separate analyses of relationships between each dormancy trait and each bioclimatic and soil variable showed that only one dormancy trait (PI_PY_) was significantly correlated with more environmental variables, while other dormancy traits were either not correlated or showed weak correlations with some environmental variables (Supplementary data Figures S2, S3 and S4, Table S4). Specifically, PI_PY_ was clearly related to the gradient of aridity, i.e., PI_PY_ increases with increasing temperatures and decreasing precipitation and decreasing available soil water capacity (Figure 2; Supplementary data Figure S2). However, there were three climatic variables, i.e., IV BIO1, IV BIO5, and IV BIO10, which showed significant correlations with a majority of dormancy traits (Supplementary data Table S5). Specifically, final PY dormancy (FPYD_M_, FPYD_25_, FPYD_35_) slightly increased with increasing inter-annual variation in temperatures of the warmest quarter (all r = ~0.19 *).

**Figure 3:**
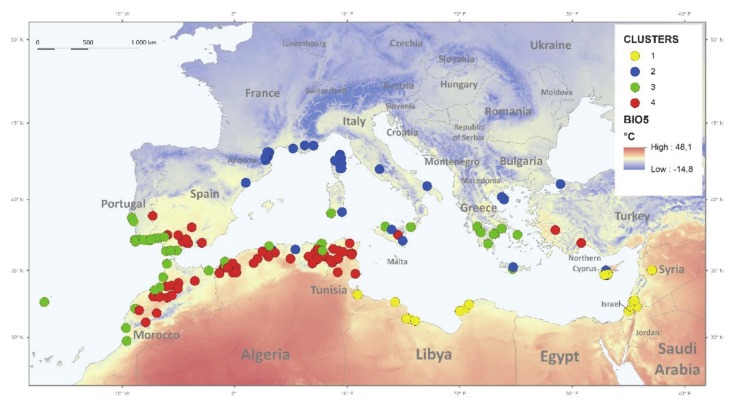
Geographic distribution of studied *Medicago truncatula* accessions classified into four clusters based on climatic and soil conditions, using Ward’s minimum-variance linkage of Euclidean distance. Grey dots indicate K1, green K2, light blue K3, and yellow K4 cluster, placed on the background of BIO5 (precipitation in the wet quarter).

**Figure 4:**
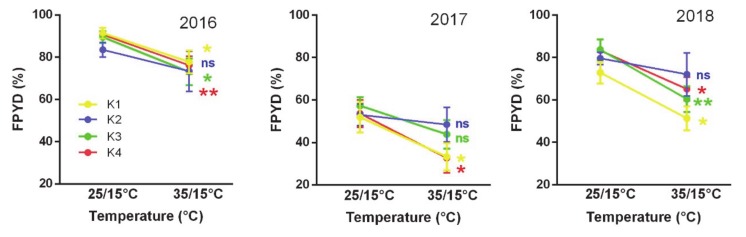
Reaction norms to changes in experimental temperature (25/15 °C, 35/15 °C treatments) on final PY dormancy of seeds for K1–K4 macro-environmental clusters in three experimental years (2016, 2017, and 2018). Vertical bars indicate ± SE. Asterisk (* *p* ≤ 0.05 and ** *p* ≤ 0.01) indicates significant differences between temperatures for each cluster.

Four macro-environmental groups of Medicago accessions (Figure 2A and Figure 3, Supplementary data Table S3) differed in slopes of the FPYD across two experimental temperature treatments (Figure 4). Considering each experimental year separately, accessions from arid conditions (clusters K1 and K4, Supplementary data Table S3) consistently showed higher FPYD at 25/15 °C and lower at 35/15 °C. In contrast, FPYD of accessions from K2 (less arid conditions) did not change significantly in response to different temperature treatments (Figure 4).

### 2.3. Association Analysis of Dormancy Traits

In order to identify molecular mechanisms underlying physical dormancy and its adaptability, we performed genome-wide association analyses for all dormancy traits (FPYD_25_, FPYD_35_, AUC_25_, AUC_35_, AUC_35-25_, PI_PY_, PI_AUC_) and three bioclimatic variables (BIO1, BIO9, BIO12) on 178 accessions. Corresponding Manhattan plots for these analyses are provided in Supplementary data Figure S5. Quantile–quantile (Q-Q) plots confirmed that FarmCPU was a more suitable model to perform association studies (Supplementary data Figure S6). Most significant Quantitative Trait Nucleotides (QTNs) were identified with AUC_25_, AUC_35-25_, FPYD_25_, PI_AUC_ and all three bioclimatic variables. To provide a list of significant QTNs, we defined a threshold of 10^−7^ (except for PI_PY_, where we used a threshold of 10^−4^). 136 candidate genes were identified as potential regulators of physical dormancy (Supplementary data Table S6). A large proportion of candidate genes was annotated as involved in synthesis of secondary metabolites, in cell wall modification, and hormone regulation. We performed an over-representation analysis with these 136 candidate genes using a hypergeometric test with Bonferroni correction and this revealed three biological functional Gene Ontology (GO) classes statistically overrepresented (Supplementary data Table S7) and acting as potential regulators of dormancy: response to oxidative stress (GO:0006979), oxidation reduction (GO:0055114), and response to chemical stimulus (GO:0042221). Candidate genes belonging to these three GO classes are indicated in Table 1.

## 3. Discussion

*Medicago truncatula* is a representative of species adapted to the typical Mediterranean climate characterized by strong seasonality with hot and dry summers often with large diurnal temperature oscillations followed by main rainfall in the autumn. In particular, the eastern and southern zones of the Mediterranean-desert transitions are associated with increased aridity [38]. Summer drought limits growth and is the major cause of seedling mortality, while winter cold limits vegetative growth. High oscillating temperatures are hypothesized [8] to be one of the main triggers of PY dormancy release and it was confirmed experimentally in several legume species, including *Lupinus, Trifolium, Pisum* [10,26,27,28] and, in this study, *Medicago*. However, these studies tested only the effect of temperature variation, while more factors vary in nature [39]. In particular, soil moisture oscillation is very difficult, if not impossible, to mimic in laboratory conditions. The effect of water potential on seed germination was tested only as a static component [40,41]. As a result, our experimental setup could reveal only temperature-related dormancy release.

### 3.1. Association between Seed Dormancy Traits and the Environment

Variation in germination strategy is particularly relevant for plants inhabiting unpredictable environments and is consistent with seed function, and securing the next generation in time and space [39]. In our study, seed dormancy release varied among accessions and years, and this could potentially act as a mechanism that favors the persistence of the seed in the soil and helps to distribute genetic diversity through time [24,42]. However, when taking average estimates of various traits characterizing absolute dormancy release (i.e., FPYD, AUC, and AUC_35-25_), no clear relationships were found between synthetic environmental (macroclimatic) clines (expressed as PCA axes) and mean dormancy status per accession (Figure 2). Our observations thus seem to be in agreement with study of Mediterranean wild lupines [29] or perennial woody legume (*Vachellia aroma*) along a precipitation gradient that did not find any relationship between climate and dormancy release [43]. In contrast, *Arabidopsis thaliana* [44,45] and other winter-annual species, such as *Betta vulgaris* subsp. *maritima, Biscutella didyma, Bromus fasciculatus*, and *Pisum sativum* subsp. *Elatius*, showed a cline in dormancy [10,43,46,47]. In particular, more dormant genotypes of mentioned taxa occurred in lower latitudes or more arid habitats with seasonally unpredictable precipitation and less dormant in higher latitudes or more humid habitats, suggesting dormancy is an adaptation securing population survival in less predictable conditions. However, in arid and unpredictable environments, there are also species called “risk-taking” with lower dormancy and rapid germination in response to lower rainfall events. The high and reliable seed production determines that the consequences of failure to establish in these species are less dire [17].

Does the absence of a relationship between average estimates of dormancy release in *Medicago* and macroclimatic clines within our dataset suggest that selection does not operate on dormancy traits? Taking into account plasticity of dormancy release between temperature treatments revealed that physical dormancy plasticity (PI_PY_) increases with increasing aridity (2D, Figure 3 and Figure 4; Supplementary data Figure S3). It follows, therefore, that more plastic behavior can potentially distribute germination across the year and act as a within-season bet-hedging strategy [48], suggesting that in more unpredictable environments genetic components of phenotypic variance may be lower and thus a reduced evolutionary response to selection would be possible [24]. The bet-hedging strategy is thus positively associated with more arid habitats as found in pea [10], and plastic responses provide potential to cope with high levels of environmental heterogeneity [49]. On the other hand, the responsiveness of the accessions of the macro-environmental clusters K1, K3 (except in 2017), and K4 to high temperatures (35/15 °C) in relation to cluster K2 (Figure 2, Figure 3 and Figure 4) could be one of the main triggers of PY dormancy release under an arid and unpredictable environment. Occasional precipitation and hot temperatures in summer would accelerate the PY dormancy release and germination after overcoming the PD dormancy. Earlier emergence can profit from a long growing season, providing a competitive and reproductive advantage for limited resources. Recently, ten Brink et al. [50] showed that more unpredictable natural environments can select earlier within-season germination phenology. In addition, we found an increase, although slight, in PY dormancy for accessions originating from sites with higher among-season temperature variations expressed by IV indexes. This could be an among-season bet-hedging strategy, which increases PY dormancy under more unstable summer temperatures between years, and might thus contribute to avoidance of increased germination under adverse temperatures condition or “false breaks” (seed germination outside the optimal growing season) [51].

### 3.2. Potential Shortcomings of the Study

Although dormancy is genetically determined, it also depends on the environmental conditions experienced by the mother plant and the subsequent status of the seed [24,39]. This was shown in several taxa, including *Trifolium* and *Medicago* [24,27,28,52,53]. There are several possible sources of variability, from the effect of the maternal plant status (drought, photoperiod, nutrition) to natural variation within a population or even the same individual [54]. Distinguishing between maternal and environmental effects is difficult. The information on the maternal environmental can be mediated via the nutrition, phytohormones, or gene expression levels and variability in the seed sensitivity [39]. All this might contribute to *M. truncatula* seed stock generated in our study. To minimize environmental maternal effects, we grew the accessions under common garden conditions (glasshouse), but these were to some extent variable between years. In 2016, the accessions were sown February to April and harvested in a hot July with a day temperature over 35 °C, while in 2017 and 2018, they were sown in September and grew over winter, flowered in February, and maturated in April, with day temperatures in the glasshouse around 28 °C. The higher temperature in 2016 during the seed filling period resulted in more dormant seeds in some accessions in relation to 2017 and 2018 (Supplementary data Figure S8). In addition to this, seeds from different accessions differ up to 3 weeks in maturation due to differences in flowering time. Moreover, the individual seed stock from a given accession was harvested during a period of about 3 weeks, which could be possibly synchronized by different sowing. This needs to be considered in follow up studies.

Our analysis was also likely impacted by several factors inherent to the available *Medicago* set. At first, there is substantial imprecision in the GPS localization of the origin of some accessions [55] leading consequently to incorrectly extracted environmental factors [56,57,58]. Secondly, there is geographical bias towards the western part of the Mediterranean with underrepresented parts of the native species range, such as Italy, Adriatic Sea coast, Turkey, Lebanon, and Israel. In addition, the characteristics of WorldClim data (averages in terms of time and also space) mask the micro-ecological pattern and, in geographically complex regions, environmental conditions change considerably over short spatial scales, such that neighboring populations can be subject to different selective pressures, as found in the study of seed dormancy of Swedish *A. thaliana* accessions [59].

### 3.3. Genetic Basis of Seed Dormancy Release in Legumes

In contrast to the seed development, the genetic basis of *Medicago* seed germination was studied only by Dias et al. [60]. These authors, however, focused on true germination, e.g., radicle emergence, removing the seed coat prior to testing and thus assessing physiological dormancy, while we were interested in PY dormancy executed by seed coat permeability. Since the dormancy is indirectly evaluated as the release from dormancy by imbibition and germination, the detected candidate genes might be related to the changes in the seed coat mediated imbibition process rather than dormancy per se. There was no overlap in associated loci between the tested seed dormancy traits, despite the detection of a similar set of candidate genes (Supplementary data Table S7). This is similar to other tested quantitative and complex traits, such as drought and biomass, where different traits had different candidate genes [61]. We have detected four genes active in flavonoid and phenylpropanoid biosynthesis pathways leading either to flavonoids or via polymerization to lignins impregnating the seed coat [12,62]. Notably, homologue genes were detected when comparing dormant and non-dormant pea seed coat expression [63]. Furthermore, hydrolytic enzymes such as xyloglucan 6-xylosyltransferase, xylogalacturonan β-1,3-xylosyltransferase involved in plant cell wall modification were identified. Notably, one of the Quantitative Trait Loci (QTL) identified in biparental mapping of *Medicago* seed germination also encodes xyloglucan endotransglucosylase [60]. The β-1,3-Glucanase (EC 3.2.1.39) plays roles in the regulation of seed germination, dormancy, and in the defense against pathogens. The β-1,3-glucan layer is in the seed coat of cucurbitaceous species and confers seed semi-permeability [64]. In tobacco seeds, β-1,3-Glucanase was shown to be at the micropylar part of the endosperm prior to radicle protrusion, and seems to play a role in cell wall loosening [65]. Pectinesterases were isolated from germinating seeds of various species and are assumed to play an important role in loosening cell walls [66]. Polygalacturonases (EC 3.2.1.15) are another cell wall degrading enzyme. These were shown to play an essential role in pollen maturation and in pectin metabolism during fruit softening and weakening of the endosperm cell walls [67]. Exo-(1→4)-β-galactanases (EC 3.2.1.23) play various roles in physiological events, including cell wall expansion and degradation during soft fruit ripening, and were found to be involved in the mobilization of polysaccharides from the cotyledon cell walls of *Lupinus angustifolius* following germination [68]. Nitric oxide (NO) was recently shown to be involved in plant development including seed germination [69]. NO-dependent protein post-translational modifications are proposed as a key mechanism underlying NO signaling during early seed germination. Our GWAS analysis identified seven putative peroxidase and thio-/peroxiredoxin genes. Peroxiredoxins (EC 1.11.1.15) catalyze the reduction of hydroperoxides, conferring resistance to oxidative stress. Recent studies have demonstrated that Reactive Oxygen Species (ROS) have key roles in the release of seed dormancy, as well as in protection from pathogens [70,71,72]. Thioredoxins were identified to promote seed germination [73]. Peroxidases (EC 1.11.1.7) are also implicated in lignin/suberin formation during the polymerization of monolignols synthetized in the final steps of the phenylpropanoid pathway [74]. Phytohormones and especially gibberellins are known to play important roles in seed development and germination (reviewed in [75]), and since *Medicago* seeds have both physical and physiological dormancy, it is not surprising to find gibberellin 20-oxidase and two ent-kaurenoic acid oxidase genes to be associated with dormancy release (AUC_25_) or environmental factors (BIO12), respectively. The genomic signature of *M. truncatula* adaptation to the climate was studied by Yoder et al. [76] using essentially the same set of lines. They analyzed the relationship to BIO1, BIO3, and BIO16; thus, there is only overlap in BIO1 (annual mean temperature) with our study. The different candidate genes were detected. This could be attributed to differences in accessions and analytical methods, as well as *Medicago* genome versions. However, despite these differences, some similar genes were detected, such as 1, 3-glucanase or kinases. Several kinases and disease resistance (TIR-NBS-LRR class) genes are associated with dormancy release traits. These have been implicated in pathogen sensing and host resistance, which might reflect the sensing of cell wall modulating enzyme activities, similar to pathogen attack [77]. As the result of bet-hedging, seeds in the soil form long-term seed banks where they need to be protected from microbial decay by presence of secondary metabolites, as well as seed defense enzymes [72,77]. Therefore, one of the possible future directions of seed dormancy release studies should be the study of seed–soil–microbiome relationships and seed coat enzymatic activities.

## 4. Materials and Methods

### 4.1. Plant Material

Seeds of *Medicago truncatula* inbred lines were selected from HapMap collection [36,76] based on accuracy of coordinates and were obtained from INRA, Montpellier, France and University of Minnesota, USA. Plants were grown in glasshouse conditions at the Department of Botany, Palacký University, Olomouc, Czechia, from March to July (2016) and from September to May (2017, 2018). Plants were cultivated in 3 L pots with sand peat substrate (1:9) mixture (Florcom Profi, BB Com Ltd., Letohrad, Czechia), watered as required, and fertilized weekly (Kristalon Plod a Květ, Agro, Czechia). Temperature varied according to weather from a minimum of 15 °C during winter to a maximum of 40 °C in late spring. Supplementary light was provided (Sylvania Grolux 600 W, Hortilux Schreder, Holland) to extend the photoperiod to 8 h during September–February and to 14 h from February to stimulate flowering. Mature pods were collected, packed in paper bags, and dried at 20 °C and 60–63% relative humidity to allow post ripening for a period of 4 to 6 weeks prior to testing. Sufficient seed stock was obtained from 178 accessions using equipment made in-house.

### 4.2. Seed Dormancy Release Experiments

Release of seed dormancy was tested as imbibition (e.g., uptake of water) and terminated when the radicle protruded the seed coat [10]. As our study was aimed to study PY release and not the germination, the values we used in all subsequent analysis correspond to imbibed seeds. To mimic natural conditions, two temperature treatments (alternating temperatures of 35/15 °C and 25/15 °C at 14/10 h (day/night) regime) were applied to intact seed batches (50 seeds, in 2 to 3 replicas per treatment). Seeds were placed onto water saturated filter papers (Whatman Grade 1, Sigma, CZ) in 60 mm Petri dishes (P-Lab, CZ) in temperature-controlled chambers (Laboratory Incubator ST4, BioTech, CZ). In order to prevent fungal growth (as seed sterilization would alter seed coat properties), fungicide (Maxim XL 035 FS; containing metalaxyl 10 g and fludioxonil 25 g) was applied. Seeds were monitored at 24 h intervals for total of 88 days. After each scoring, the plates were randomly relocated within chambers. Germinated seeds (e.g., when the radicle protruded from the testa) were removed. At the end of the testing, remaining seeds were scarified and let germinate to verify their viability. This typically was over 98%. Although we selected macroscopically intact seeds for experiments, we cannot exclude some microscopic cracks on seeds resulting from mechanical damage during threshing. We observed that in the course of the first hours of seed germination experiments, a certain proportion of the seeds imbibe. Therefore, we subtracted the first day imbibition value from the analysis. In 2016, 2017, and 2018, a total of 147, 74, and 130 accessions were tested (Supplementary data Figure S8). In total, seeds of 178 accessions were included in the experiments (Supplementary data Table S1). Forty-seven accessions were tested in all three years, and 129 accessions in at least two years.

### 4.3. Evaluation of Dormancy and Germination Traits

Several statistics (traits) characterizing dynamics and final state of dormancy release of seeds of each accession for each treatment (i.e., 25/15 °C and 35/15 °C) were calculated as follows: *(i)* Final PY dormancy (%; FPYD_25_, FPYD_35_): represents percentage of dormant seeds at the end of experiment after excluding seeds germinating during the first day of each experiment (i.e., 100 – final germination percentage after 88 days + germination percentage after first day), calculated separately for two germination treatments. *(ii)* Germination pattern (AUC_25_, AUC_35_): this trait represents the area under curve (AUC) coefficient that takes into account both dynamics of germination as well as final germination percentage. Original germination data (daily counts of imbibed seeds) were considered as discrete realizations of an asymptotically continuous process, approximated by spline functions [78,79]. The resulting smoothing spline, called the absolute germination distribution function (AGDF; as applied in pea [10]), was used in analysis. Accordingly, the area under curve (AUC) of the spline function takes into account both the course of the germination as well as the final score of germinated seeds, which captures the dynamics of seed germination better than existing germination coefficients [80,81]. High AUC values mean rapid and early germination of the majority of seeds. *(iii)* FPYD_M_ and AUC_M_: these two coefficients represent means of respective coefficients estimated for the two temperature treatments (e.g., FPYD_M_ = (FPYD_25_ + FPYD_35_)/2)). *(iv)* Germination response (AUC_35-25_): this is the difference between two AUC (i.e., AUC_35_ − AUC_25_) of each accession calculated for two germination treatments (i.e., 35/15 °C and 25/15 °C). Higher absolute values of germination response mean larger differences in germination pattern of the same accession between germination treatments, while the sign of the difference suggests which of the treatment shows the larger AUC. *(v)* Phenotypic plasticity index of the germination pattern (PI_AUC_) and *(vi)* Phenotypic plasticity index of final PY dormancy (PI_PY_): these traits were calculated for each accession as (trait_max_ − trait_min_)/trait_max_, where trait_max_ and trait_min_ were, respectively, the maximal and minimal value of the trait measured on the same accession across the two temperature treatments (25/15 °C and 35/15 °C). These estimates characterize the maximal plastic capacity of an individual in variable environments without taking into account the direction of the plastic response or the change in intensity with environmental variation. Phenotypic plasticity index ranges from 0 (no plasticity) to 1 (maximal plasticity) [2]. For the purpose of multivariate analyses, we calculated the average of each dormancy trait for each accession over the years. Consequently, the matrix of averages of each dormancy trait for each accession was used in multivariate analyses. Multicollinearity among variables was assessed by the variance inflation factor (VIF) for quantitative traits using the library usdm in R [82]. Only variables whose VIF was lower than 15 were retained in the analyses. Except for FPYD_M_ and AUC_M_, none of the above-mentioned traits had a collinearity problem.

### 4.4. Extraction of Environmental Variables and Spatial Accuracy

Due to different spatial accuracies of accessions and in order to minimize the spatial error caused by imprecise coordinates, we developed a geoprocessing model in the ArcGIS PRO environment [83]. The model automated the calculation of mean values of selected variables from within a 5 km buffer around each collection site in order to smooth the uncertainty caused by imprecise localization. The WorldClim database version 2.0 [84] was used to extract climatic data (period 1970–2000) from GeoTIFF rasters in the WGS-84 coordinate system (EPSG: 4326) with a spatial resolution of 30 arc-seconds (~1 km). Bioclimatic variables (BIO1–BIO19) were derived from the monthly temperature and rainfall values [85], and represent annual trends (e.g., mean annual temperature BIO1, annual precipitation BIO12), seasonality (e.g., annual range in temperature and precipitation BIO4 and BIO15) and extreme or limiting environmental factors (e.g., the temperature of the coldest and warmest month BIO5 and BIO6, and amount of precipitation in the wet and dry quarters BIO16 and BIO17). In order to determine the inter-annual variability in selected bioclimatic variables (BIO1, BIO5, BIO10, and BIO12) during the period 1981–2010, the index of variability (IV) was calculated following the percentile-analysis method [86]. To obtain yearly mean values for years 1981–2010, we used 2 m air temperature (Kelvin degrees) and 2 m specific humidity (kg of water/kg of air) hourly data from the Modern Era Retrospective Analysis for Research and Applications Reanalysis (MERRA) 2D Incremental Analysis Update atmospheric single-level diagnostics product (MAT1NXSLV), provided by the NASA Global Modelling and Assimilation Office [87]. Data were interpolated in spatial resolution of 2.5 arc-minutes. Temperature data were converted to degrees of Celsius (BIO1, 5, and 10). The resulting BIO12 (1981–2010) describes the annual mean of specific humidity instead of cumulative annual rainfall.

Twelve month means (BIO1, 5, 10, and 12) for each site were calculated as follows:

IV = [(90th percentile − 10th percentile)/50th percentile] ∗ 10(1)

The different IV classes are low (IV <0.50), low–moderate (0.50–0.75), moderate (0.75–1.00), moderate–high (1.00–1.25), high (1.25–1.50), very high (1.50–2.00), and extreme (IV >2.00).

Soil data were extracted from the SoilGrids database [88]. SoilGrids prediction models are fitted using over 230,000 soil profile observations from the World Soil Information Service (WoSIS)database and a series of environmental covariates. Covariates were selected from environmental layers from Earth observation-derived products and other environmental information, including climate, land cover and terrain morphology [89].

### 4.5. Climate and Soil Characteristics of Localities of Studied Accessions

The set of accessions originates from rather contrasting climatic conditions (Supplementary data Table S2). Mean annual temperature ranges from ca 9 to 22 °C and annual precipitation ranges from 154 to 1028 mm; consequently, temperature annual range (min–max) is from 13 to 35 °C. Some accessions originate from sites with minimal winter temperatures below zero while maximal temperature of warmest months is rather similar among accessions. However, sites differ considerably in precipitations of driest and warmest periods. The basic descriptive statistics of the index of variability (IV) for BIO1, BIO5, BIO10, and BIO 12 are present in Table S2. IV BIO1 ranged from 0.33 to 1.60 with mean 0.77 (SD = 0.20), IV BIO5 from 0.57 to 2.26 with mean 1.01 (SD = 0.32), IV BIO10 from 0.63 to 1.76 with mean 0.98 (SD = 0.20), and IV BIO12 from 0.46 to 2.03 with mean 0.96 (SD = 0.28). The most variable IV was IV BIO5 (range 1.69) followed by IV BIO12 (range 1.57). Concerning soil variables, considerable variability among sites was found in volumetric percentage of coarse fragments (CRFVOL) and soil organic carbon content (ORCDRC), while other soil variables were more consistent among sites (Supplementary data Table S2).

### 4.6. Testing of Relationships Among Dormancy Traits, Geography and Environmental Variables

The matrix of environmental variables was checked for the presence of the multicollinearity using VIF. The reduced set of environmental variables (with VIF <15), including 14 bioclimatic variables and eight soil variables, was used in all further analyses. For each pair of variables, bivariate scatter plots together with fitted locally weighted smoothing were displayed and Pearson’s Correlation Coefficient was calculated using the library Performance Analytics in R [90].

The matrix of the reduced set of environmental variables was analyzed by principal component analysis (PCA; [91]) using Canoco 5.10 [92] to find the main environmental gradients within the dataset. Several precipitation variables were log(x+1) transformed and subsequently each variable was standardized to zero mean and unit variance before PCA. A set of germination traits and geographic coordinates (latitude, longitude) were used as supplementary variables and correlated with the first two principal components. To control for possible spatial autocorrelation between each germination trait and principal components representing environmental gradients, a modified version of the *t*-test [93] was performed in SAM 4.0 (Rangel et al., 2010) [94]. To assess whether there is spatial autocorrelation present in the PCA scores along the first two axes and dormancy traits, Moran’s I spatial correlation statistic [88] was calculated for each variable using PASSaGE v. 2.0 [95]. Ten distance classes with equal widths were created and Moran’s I and its 95% CI were calculated for each distance class.

### 4.7. Phenotypic Plasticity by Macro-Environmental Clusters

We used accessions that had been tested over 3 years (Supplementary data Table S1) to calculate a norm of reaction for final PY dormancy (FPYD_25_ and FPYD_35_). First, selected *Medicago* accessions were grouped into four macro-environmental clusters (Supplementary data Table S3) based on Euclidean distance of environmental variables used for calculations of PCA. Agglomeration was performed using Ward’s minimum-variance linkage algorithm. Before clustering, all variables were standardized to zero mean and unit variance. Second, a norm of reaction for each macro-environmental cluster was estimated as follows: each line represents the data for a different cluster and the effect of “environment” (treatments, 25/15 °C and 35/15 °C), separately for each experimental year [2]. To focus on the change of the trait in response to two temperature treatments we analyzed the FPYD means within each cluster per each year by ANOVA using the InfoStat software [96].

### 4.8. Genome-Wide Association Analysis

Genome-wide association analysis was performed on seven seed dormancy traits (FPYD_25_, FPYD_35_, AUC_25_, AUC_35_, AUC_35-25_, PI_PY_, PI_AUC_) and three bioclimatic variables (BIO1, BIO9, BIO12) on 178 accessions. Prior to GWA analyses, normal distribution of each trait was checked using the Shapiro–Wilk test. Two contrasted algorithms were used to test markers–traits associations: Efficient Mixed Model Association (EMMA), a classical mixed linear model (MLM) for single locus GWAS [97], and FarmCPU, a multi-locus method combining the fixed effect model and random effect model iteratively in order to improve the statistical power of MLM methods [98]. Both algorithms were implemented in the R package rMVP using default parameters (P-value threshold 0.01) and run using a Single Nucleotide Polymorphism (SNP) dataset containing 5.85 million SNPs remapped in the *Medicago* genome v.5 [99]. Population structure, calculated using STRUCTURE by Bonhomme et al. [100], was used as a covariable. Normal distribution, QQ plots, and single/multiple Manhattan plots were performed using R package rMVP. *Medicago* genome version 5.0 of A17 genotype [101] was used to search for the encoded genes within the region of 10 kb from detected SNP. Transposable elements were excluded from the search. To link identified QTNs with putative causal gene by considering the linkage disequilibrium (LD), we selected all SNPs correlated (r^2^ > 0.7) with the top identified QTNs within a 15kb genomic range, corresponding to the average LD block size present in the *Medicago* Hapmap population [100,102], and we listed gene IDs closely related to these SNPs. Seed expression pattern of the candidate genes was assessed using published *Medicago* seeds or seed coat expression studies [103,104] and web-based expression atlas [105]

## 5. Conclusions

We found that phenotypic plasticity of seed dormancy release was significantly correlated with increased gradient of aridity, suggesting that plastic responses to external stimuli provide seeds with bet-hedging capacity and the potential to cope with high levels of environmental heterogeneity. Genome-wide association analysis identified candidate genes associated with dormancy release. Gene ontology showed enrichment for genes involved in modification of the cell wall, as well as oxidative stress protection, mediating seed coat permeability and, ultimately, imbibition and germination. Knowledge of the seed dormancy regulation by environmental factors could be extended to other legume species, particularly to crop wild relatives of economically important species, such as chickpea, lentil, faba bean and soybean, as well as used in the management of endangered plant species with physical seed dormancy.

## Figures and Tables

**Table 1:** List of Quantitative Trait Nucleotides (QTNs) identified by genome-wide association (GWA) analysis of each dormancy trait and belonging to one of three biological function over-represented in our complete list of candidate QTNs. Corresponding chromosome locations and p-values of QTNs are indicated as well as the closest gene ID within +/- 10 kb genomic interval and its corresponding annotations (Mtv5 annotations, Mtv4 annotations, gene ontology, and gene description). For complete list and description of all identified QTNs see Supplementary data Tables S6 and S7.

Chrom	Position of QTN	P-value	Candidate gene ID within +/- 10kb interval (Mtv5)	Gene ID v4	Gene annotation	Gene description
**1**	34656783	4.25 × 10^−11^	MtrunA17Chr1g0184131	Medtr1g070110	Hyoscyamine 6-dioxygenase	secondary metabolism.flavonoids.dihydroflavonols
**1**	49585766	1.36 × 10^−7^	MtrunA17Chr1g0204011	Medtr1g101830	Peroxidase	misc.peroxidases
**1**	50761951	2.79 × 10^−7^	MtrunA17Chr1g0205571	Medtr1g104590	Primary-amine oxidase	misc.oxidases - copper, flavone etc
**1**	50761951	4.23 × 10^−8^	MtrunA17Chr1g0205541	Medtr1g104550	Primary-amine oxidase	misc.oxidases - copper, flavone etc
**2**	10895258	5.60 × 10^−9^	MtrunA17Chr2g0291751	Medtr2g028980	Peroxidase	misc.peroxidases
**2**	12184616	5.76 × 10^−9^	MtrunA17Chr2g0293411	Medtr2g031920	Ent-kaurenoic acid oxidase 2	hormone metabolism.gibberelin.synthesis-degradation.ent-kaurenoic acid hydroxylase/oxygenase
**4**	53502844	5.22 × 10^−8^	MtrunA17Chr4g0061311	Medtr4g109360	UDP-glucose 6-dehydrogenase	cell wall.precursor synthesis.UDP-Glc dehydrogenase (UGD)
**4**	53502844	3.62 × 10^−8^	MtrunA17Chr4g0061381	Medtr4g109470	Flavonoid 3’-monooxygenase	secondary metabolism.flavonoids.dihydroflavonols.flavonoid 3-monooxygenase
**4**	61245281	2.56 × 10^−7^	MtrunA17Chr4g0072091	Medtr4g127670	Peroxidase	misc.peroxidases
**5**	4768980	2.23 × 10^−12^	MtrunA17Chr5g0400421	Medtr5g014250	Beta-amyrin 11-oxidase-like	hormone metabolism.gibberelin.synthesis-degradation.ent-kaurenoic acid hydroxylase/oxygenase
**5**	33045218	2.41 × 10^−7^	MtrunA17Chr5g0432331	Medtr5g074710	Peroxidase	misc.peroxidases
**5**	33045218	2.41 × 10^−7^	MtrunA17Chr5g0432361	Medtr5g074770	Peroxidase	misc.peroxidases
**6**	28332981	4.21 × 10^−10^	MtrunA17Chr6g0474391	Medtr6g464620	Gibberellin 3-beta-dioxygenase	hormone metabolism.gibberelin.synthesis-degradation.GA20 oxidase
**6**	34330903	7.87 × 10^−6^	MtrunA17Chr6g0479681	Medtr6g072490	Cytokinin hydroxylase-like	misc.cytochrome P450
**8**	6442861	1.76 × 10^−9^	MtrunA17Chr8g0343001	Medtr8g018650	Seed linoleate 9S-lipoxygenase	hormone metabolism.jasmonate.synthesis-degradation.lipoxygenase
**8**	48706047	1.32 × 10^−9^	MtrunA17Chr8g0391921	Medtr8g105630	Glutathione peroxidase	redox.ascorbate and glutathione.glutathione

## Supplementary Materials

The following are available online at https://www.mdpi.com/2223-7747/9/4/503/s1, Figure S1: Frequency distributions of dormancy traits, Figure S2: Correlation between the phenotypic plasticity index of final dormancy (PIPY) and selected environmental variables, Figure S3: Correlation chart of the dormancy release traits and environmental variables, Figure S4: Correlation chart of the dormancy release traits, soil variables and inter-annual climatic variables, Figure S5: Manhattan plots of mapped SNP markers associated with dormancy or bioclimatic traits, Figure S6: Quantile–quantile (Q-Q) plots for all the traits obtained by standard mixed linear model (EMMA) and multi-locus linear model (FarmCPU), Figure S7: Geographic distribution of studied M. truncatula accessions, Figure S8: Final PY dormancy (FPYD) of each year under two temperature treatments (35/15 °C and 25/15 °C). Different letters indicate significant differences among year for each temperature (Fisher’s LSD test, α = 0.05), Table S1: List of tested Medicago accessions with calculated seed dormancy traits and extracted environmental variables, Table S2: Basic descriptive statistics of 23 bioclimatic variables and 10 soil variables of sites of accessions origin, Table S3: Classification of 176 M. truncatula accessions in four cluster based on environmental and climatic conditions, Table S4: Pearson coefficients-probabilities between dormancy traits and bioclimatic variables, Table S5: Regression coefficient (r2) between environmental variables and plasticity index by macroecological and genetic clusters [106], Table S6: Complete list of QTN identified by GWA studies for each dormancy trait, Table S7: Over-representation analysis of the 136 candidate genes potentially involved in dormancy traits.

## Abbreviations

PY	physical dormancy
FPYD	final PY dormancy at 25/15 °C and 35/15 °C (FPYD_25_, FPYD_35_)
AUC	area under curve representing germination pattern (AUC_25_, AUC_35_)
PI_AUC_	phenotypic plasticity index of the germination pattern
PI_PY_	phenotypic plasticity index of final PY dormancy
IV	index of variability
FPYD_M_	means of FPYD coefficients estimated for the two temperature treatments
AUC_M_	means of AUC coefficients estimated for the two temperature treatments
