# Supplementary material for: Physical Dormancy Release in Medicago truncatula Seeds Is Related to Environmental Variations
Source: Plants (Basel). 2020 Apr 14;9(4):503. doi: 10.3390/plants9040503 (PMC7238229; doi:10.3390/plants9040503)
Supplement: Supplementary file 1 [file plants-09-00503-s001.zip › FigS5.pdf]

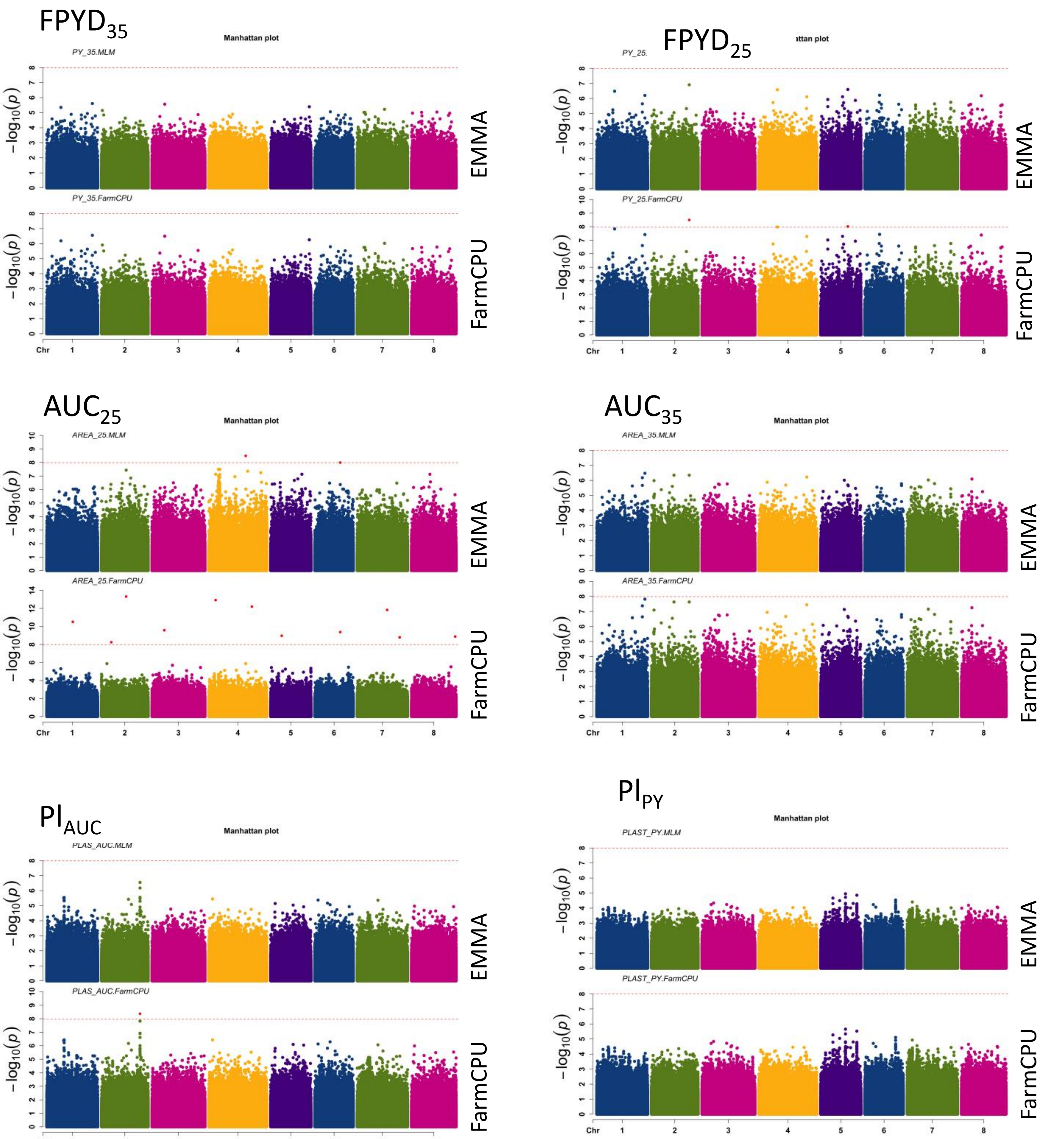

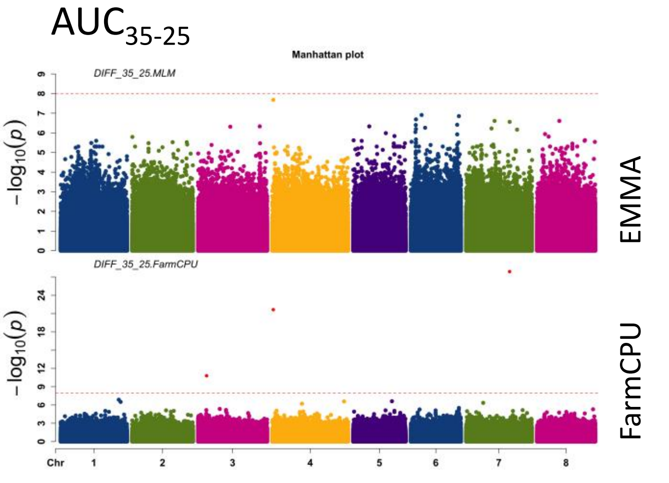

Figure 7S : Manhattan plots (comparison EMMA versus FarmCPU) of mapped SNP markers associating with each dormancy trait. Chromosomes and SNP positions on chromosome are mapped on the x-axis, -log10(p-values) are mapped on the y-axis

BIO_1

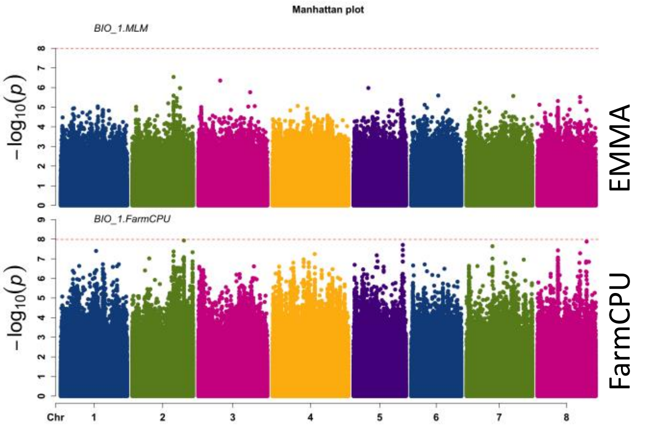

BIO_9

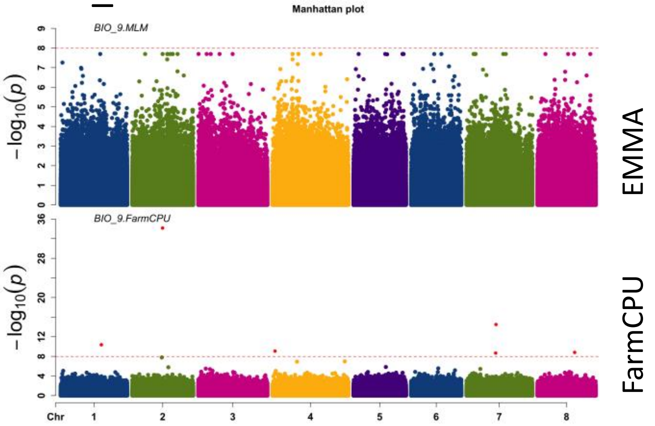

BIO_12

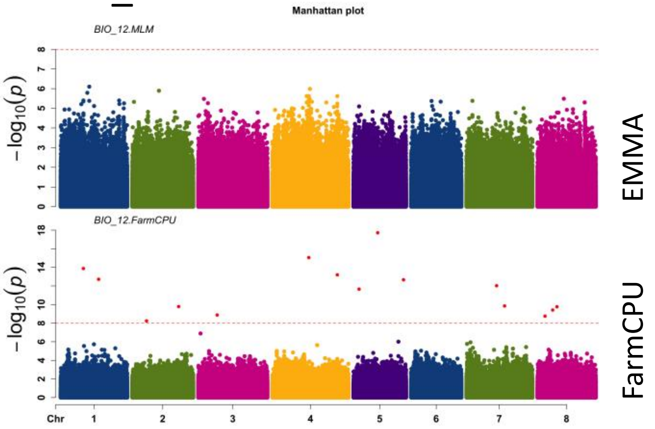

Figure 7S : Manhattan plots (comparison EMMA versus FarmCPU) of mapped SNP markers associating with each bioclimatic trait. Chromosomes and SNP positions on chromosome are mapped on the x-axis, -log10(p-values) are mapped on the y-axis
