# Supplementary material for: Physical Dormancy Release in Medicago truncatula Seeds Is Related to Environmental Variations
Source: Plants (Basel). 2020 Apr 14;9(4):503. doi: 10.3390/plants9040503 (PMC7238229; doi:10.3390/plants9040503)
Supplement: Supplementary file 1 [file plants-09-00503-s001.zip › FigS6.pdf]

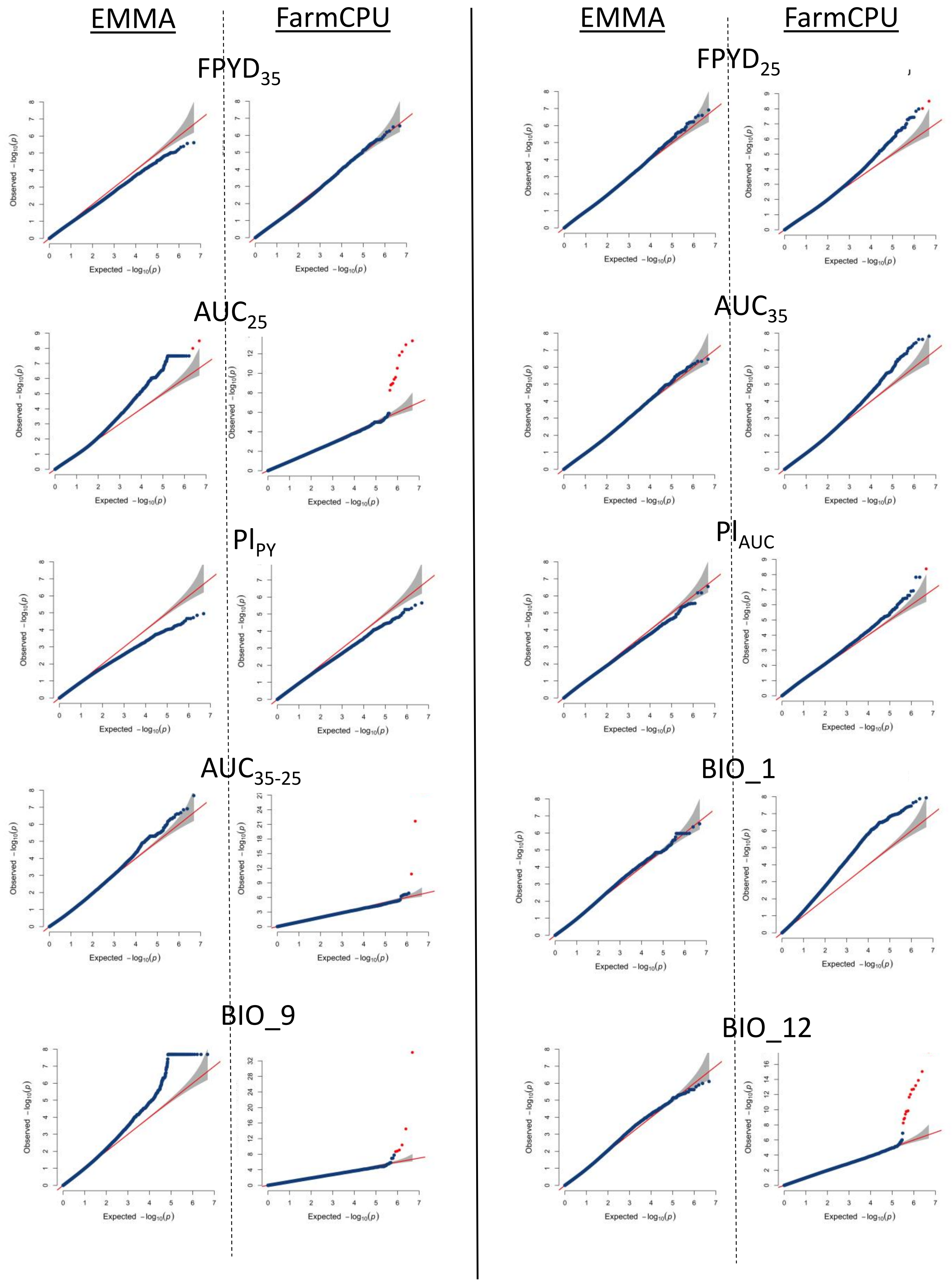

Figure 8S : Quantile–quantile (Q-Q) plots for all the traits obtained by standard mixed linear model (EMMA) and multi-locus linear model (FarmCPU). Observed $–\log_{10}$(p-value) on the y-axis and Expected $–\log_{10}$(p-value) on the x-axis.
