# Supplementary figures and images for: Physical Dormancy Release in Medicago truncatula Seeds Is Related to Environmental Variations
Source: Plants (Basel). 2020 Apr 14;9(4):503. doi: 10.3390/plants9040503 (PMC7238229; doi:10.3390/plants9040503)

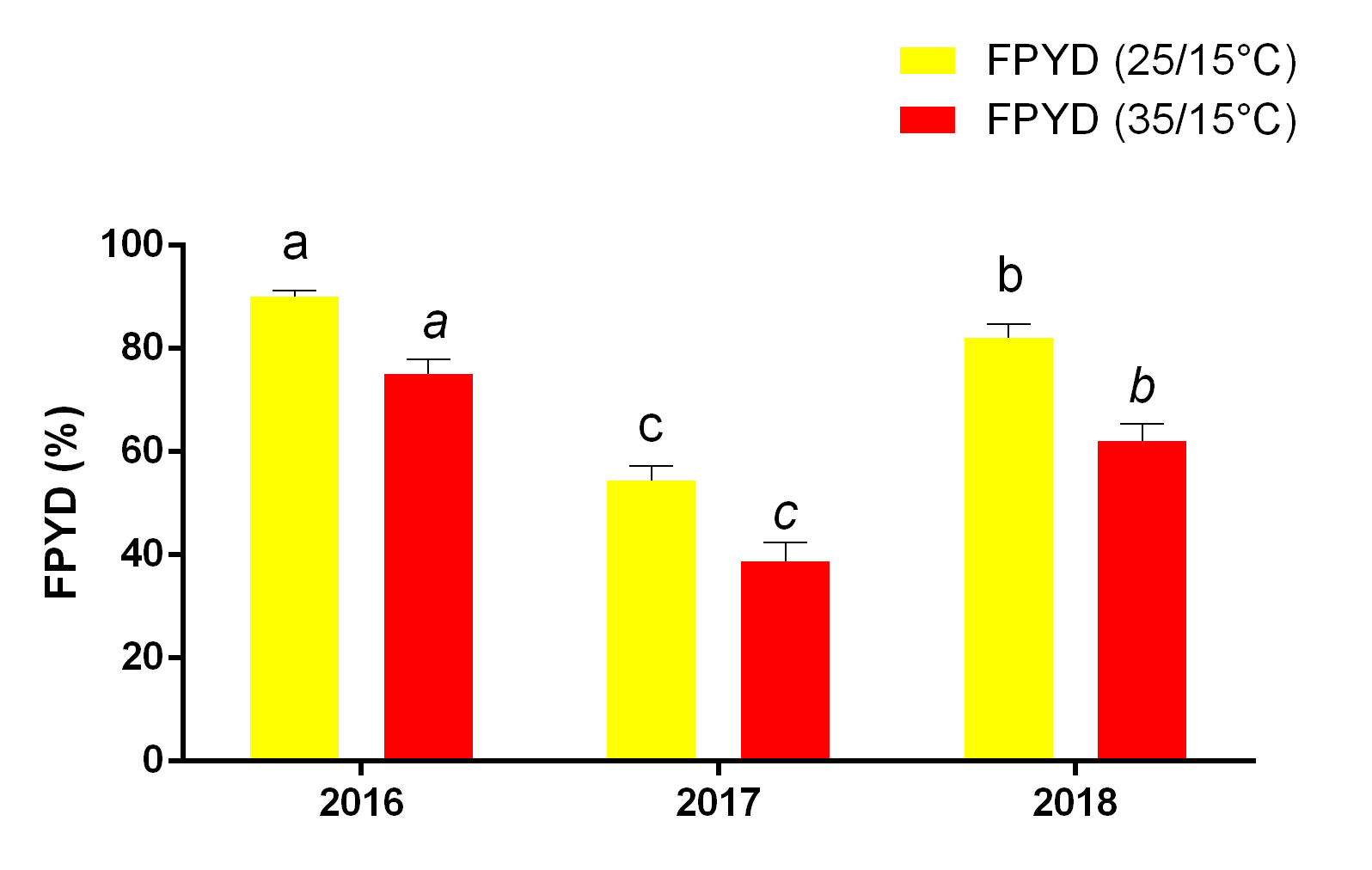

Supplement: Supplementary file 1 [file plants-09-00503-s001.zip › Fig 8S.jpg]

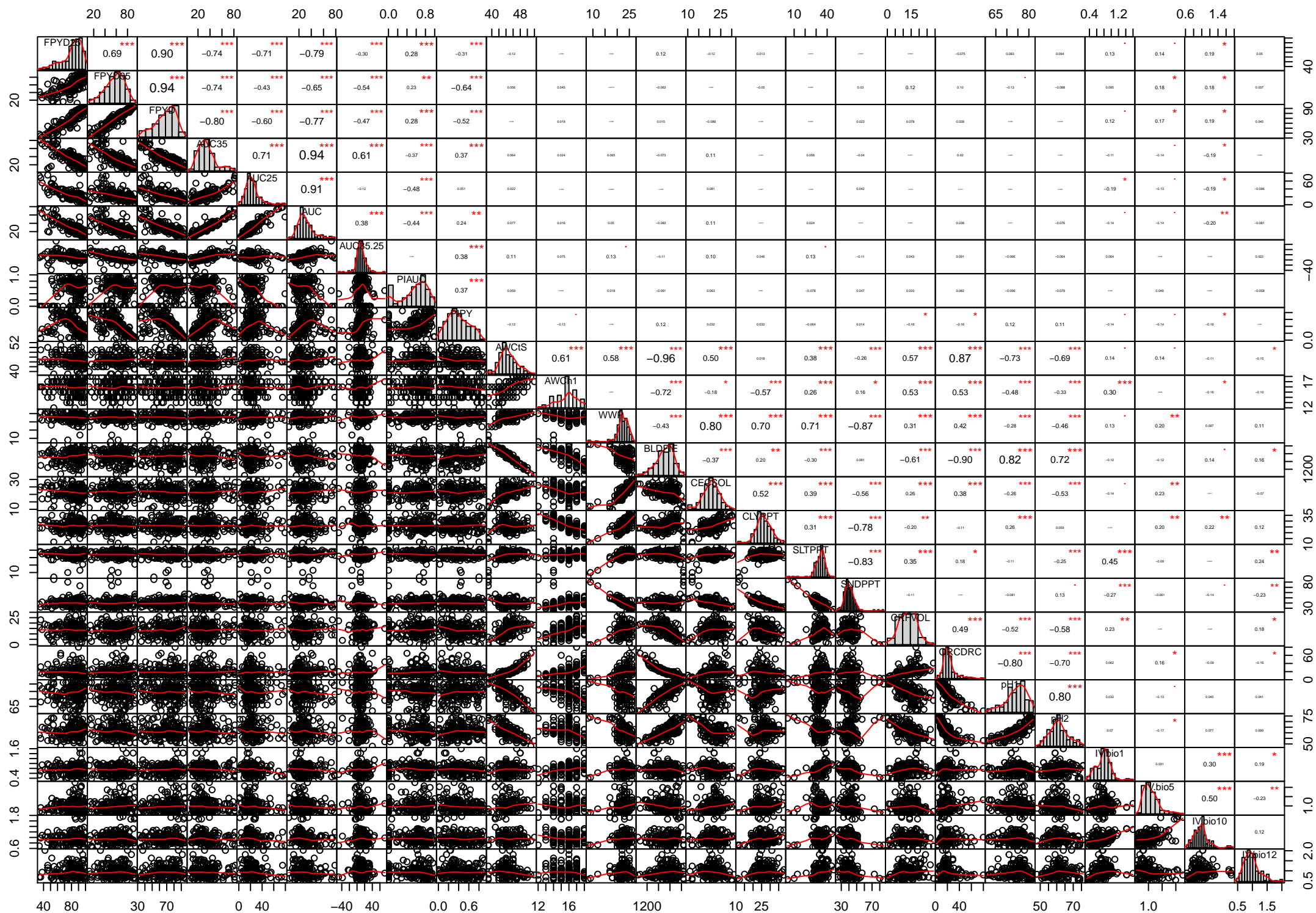

Supplement: Supplementary file 1 [file plants-09-00503-s001.zip › Fig.S4.pdf]

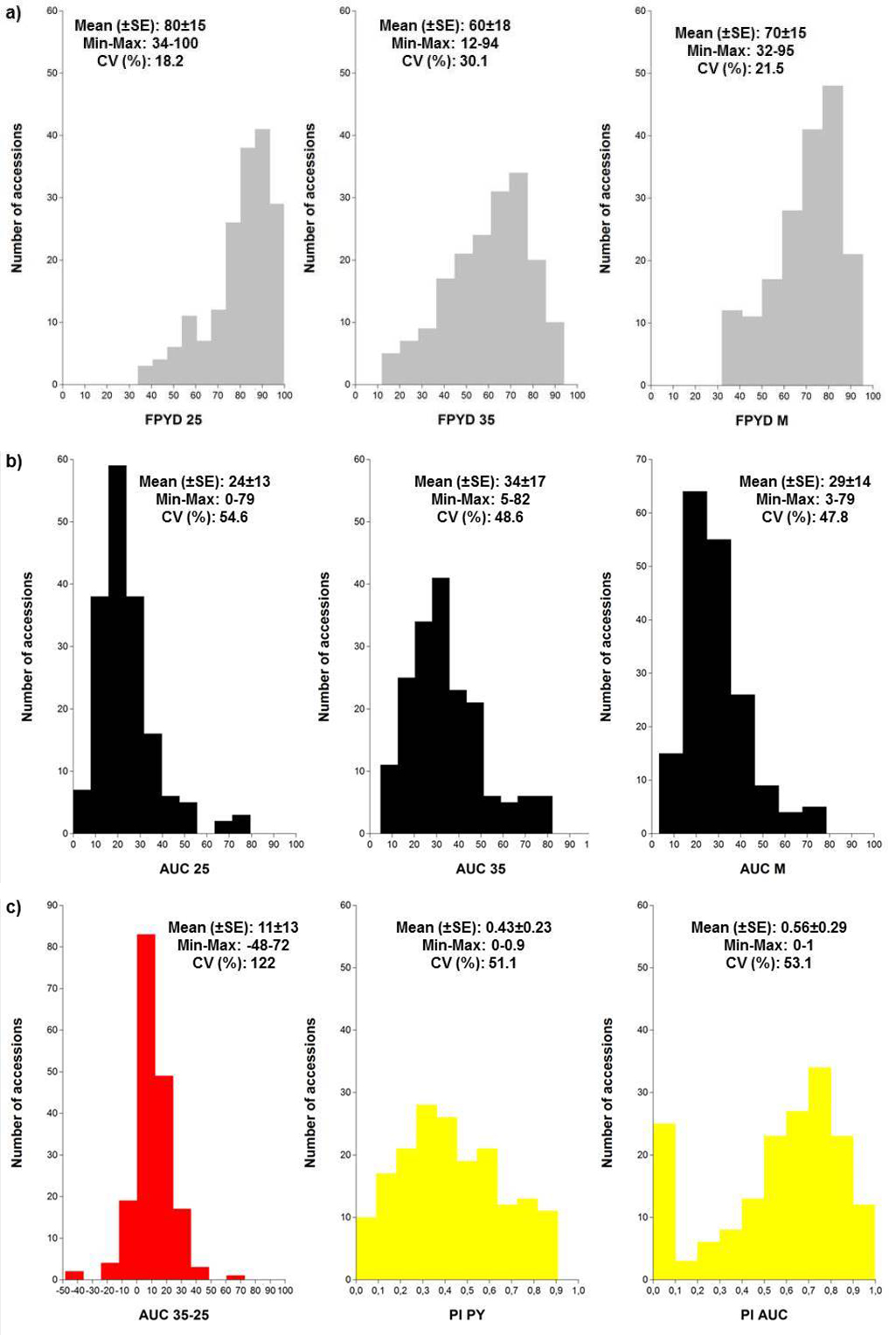

Supplement: Supplementary file 1 [file plants-09-00503-s001.zip › FigS1.jpg]

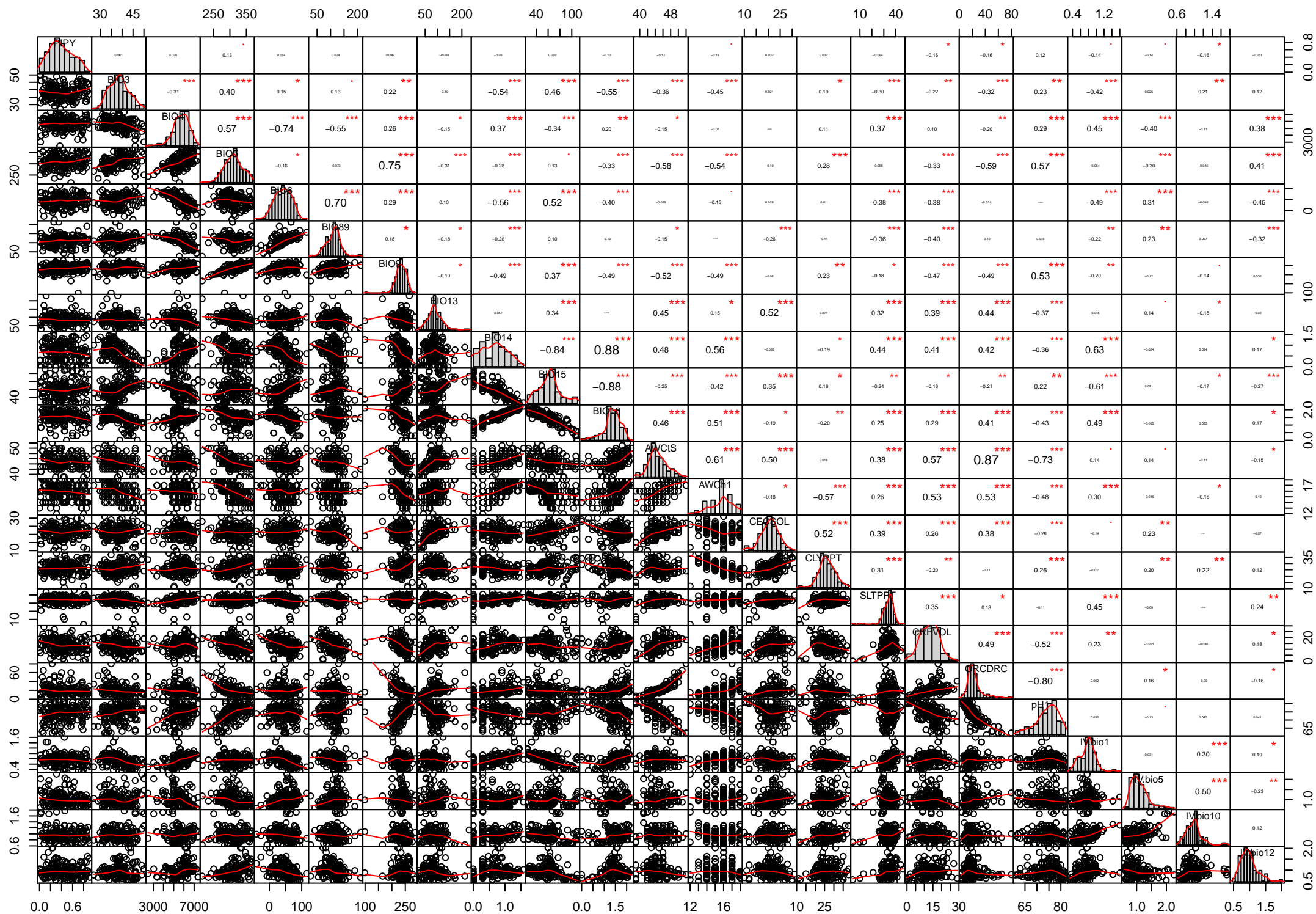

Supplement: Supplementary file 1 [file plants-09-00503-s001.zip › FigS2.pdf]

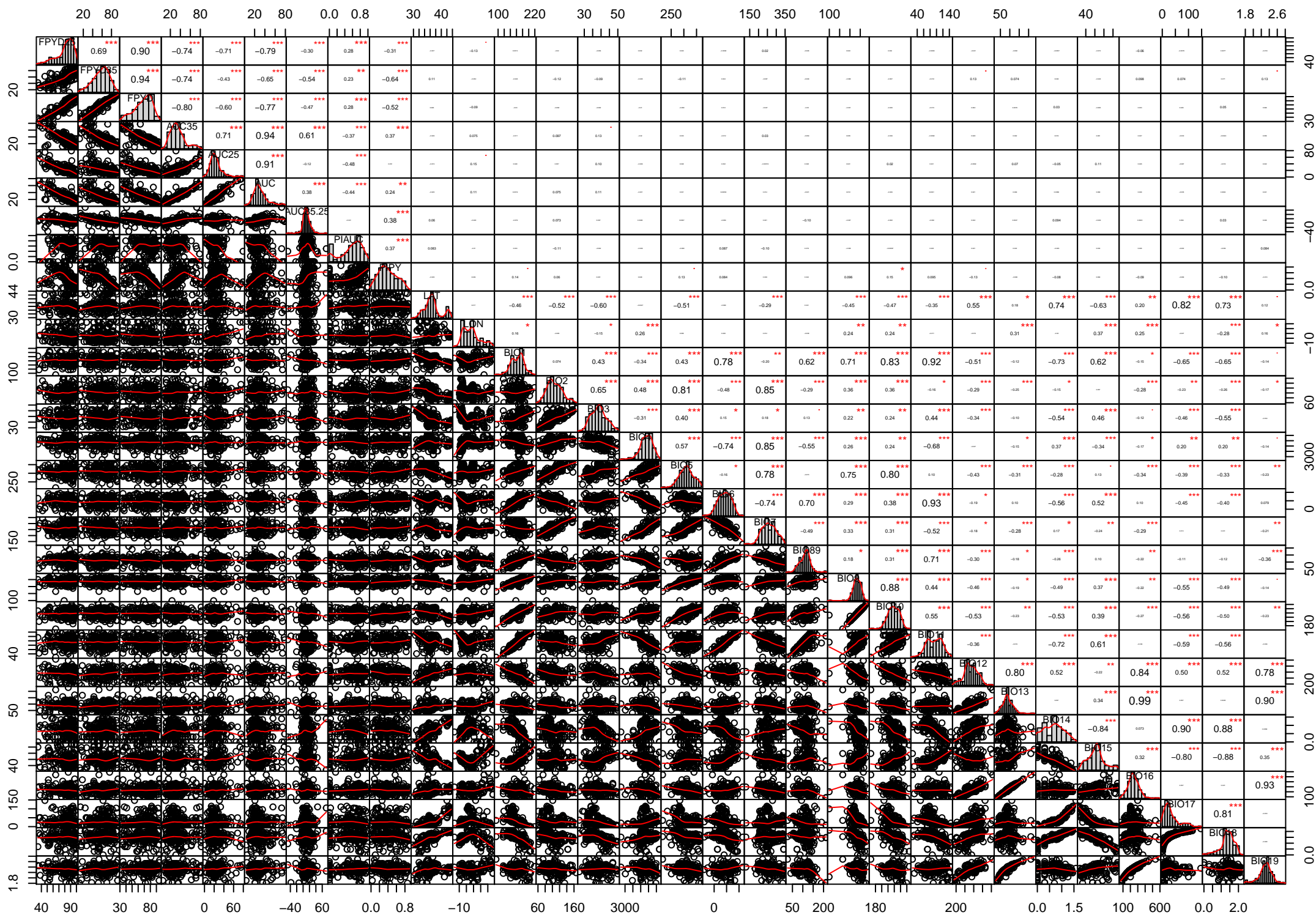

Supplement: Supplementary file 1 [file plants-09-00503-s001.zip › FigS3.pdf]

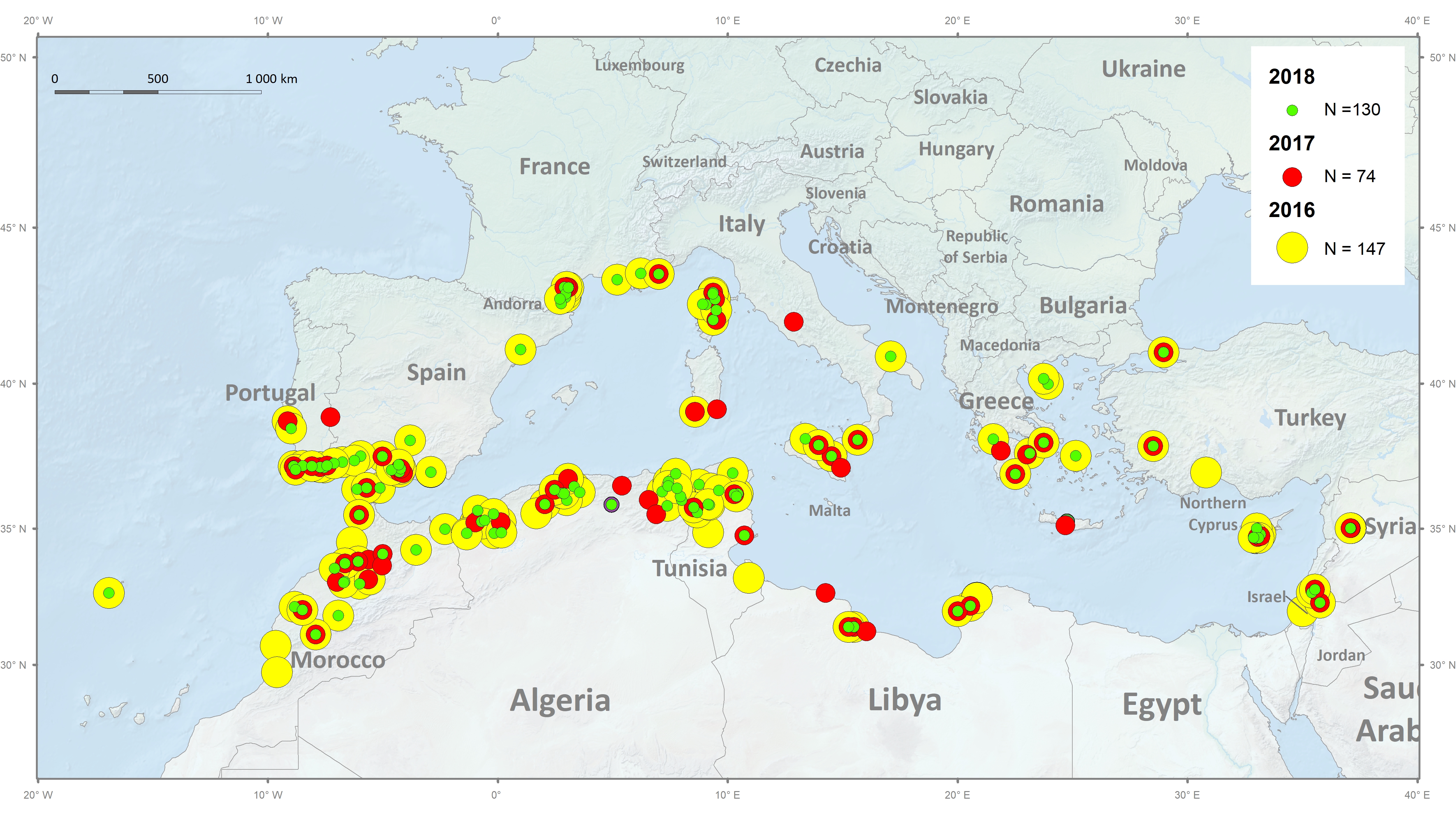

Supplement: Supplementary file 1 [file plants-09-00503-s001.zip › FigS7.jpg]
